# Cocreating a Digital Patient Preparedness Tool for International Students Accessing Primary Care in Germany: A Convergent Methods Study Within the Health CASCADE Network

**DOI:** 10.2196/87519

**Published:** 2026-09-14

**Authors:** Vinayak Anand Kumar, Margrit Schreier, Maria Giné-Garriga, Maria Ortmann, Fatima-Zohra Belmokhtar, Simona Grineviciute, Tran Ngoc-Huong Quan, Likhita Aluru, Sonia Lippke

**Affiliations:** 1 School of Business, Social and Decision Science, Constructor University, Campus Ring 1, Bremen, 28759, Germany, 49 421 200 4730; 2 Department of Psychology, University of Bamberg, Bamberg, Germany; 3 School of Health Sciences Blanquerna, Universitat Ramon Llull, Barcelona, Catalonia, Spain; 4 Faculty 11 Human and Health Sciences, University of Bremen, Bremen, Germany; 5 Department of Neurobiology, Northwestern University, Evanston, IL, United States; 6 Faculty of Health, Hamburg University of Applied Sciences/ Hochschule für Angewandte Wissenschaften, Hamburg, Germany

**Keywords:** digital health, cocreation, behavior change, patient preparedness, primary care, international students, communication barriers, behavior change wheel, COM-B model, Germany, Capability, Opportunity, Motivation – Behavior Model

## Abstract

**Background:**

Digital health interventions (DHIs) are a potential tool to address communication challenges in primary care medicine by improving patient participation, treatment adherence, and self-management of chronic diseases. However, Germany’s digital health infrastructure remains underdeveloped compared to other Organization for Economic Cooperation and Development (OECD) countries. Designing tailored, context-sensitive tools requires a deep understanding of patient needs, particularly for underserved populations navigating complex health care systems. Participatory approaches that actively involve end-users and stakeholders can help ensure that digital health tools are responsive to these needs. Behavioral science frameworks such as the Behavior Change Wheel (BCW), which incorporates the Capability, Opportunity, Motivation–Behavior (COM-B) model, can further support this process. Such technologies, developed using cocreation and behavior change frameworks, may improve health outcomes for underserved patient populations by addressing context-specific needs. This study explores how cocreation and behavioral science can inform the adaptation of a digital patient preparedness tool for international students accessing primary care in Germany.

**Objective:**

This study aimed to gain insight into international students’ experiences with primary care in Germany and explore whether adapting an existing digital patient preparedness intervention could address communication challenges and improve the standard of care. Using cocreation methods and the Behavior Change Wheel (BCW), we identified key design specifications and behavior change levers to inform tool development.

**Methods:**

A mixed methods design was used to identify design specifications for a DHI across 4 cocreation workshops with 12 students at an international university in Germany. Quantitative data were used for descriptive insights, and qualitative data were analyzed using qualitative content analysis. Workshops were informed by the BCW and the Health CASCADE cocreation methods selector tool.

**Results:**

Cocreators reported feeling misunderstood, anxious, and ill-informed during primary care interactions, with system-level barriers compounding communication difficulties. Despite this, many engaged in preparatory behaviors (eg, note-taking) to manage uncertainty and structure their consultations. Feedback on an existing digital intervention was mixed: while cocreators appreciated its intent, structured lesson formats were seen as too time-consuming. Cocreators preferred a concise, interactive design. Communication prompts, appointment scheduling, and personalized feedback were frequently requested features, though tool adoption was seen as contingent on addressing broader system-level frustrations.

**Conclusions:**

International students’ negative health care experiences, often stemming from unclear communication, perceived indifference, and difficulty navigating an unfamiliar medical system, may be mitigated through codesigned, personalized digital interventions. Frequently requested features, such as appointment scheduling, clinic directories, test result access, and interactive tools like chatbots, may help address barriers related to system navigation, communication, and access. This study demonstrates how cocreation methods can be integrated with the BCW to inform the development of context-sensitive digital health tools for specific target groups.

## Introduction

Effective communication between patients and practitioners is pivotal to the quality and delivery of health care services [1]. This is especially true in primary care medicine where physicians serve as trusted advisors, functioning as the central hub for managing complex, chronic conditions and navigating care between multiple specialists. In these care spaces, effective patient-physician communication was found to improve understanding, trust, and engagement in health-related behaviors [2,3]. However, the burden on the primary care system in Germany, including physician shortages and resource constraints [4-6], has given rise to communication failures, which in turn have implications for the perceived quality of care [7,8]. These challenges are particularly pronounced for migrant populations in Germany, who are unfamiliar with the nation’s health care system and clinical care methodology [9]. Communication challenges in German health care services, such as language and cultural barriers [10], have been linked to poor symptom management [11] and worsened patient outcomes [12].

Digital health interventions (DHIs) could be practical tools that address the communication challenges in primary care settings and improve overall standard of care. For example, in Norway, a digital symptom-reporting and communication intervention was shown to enhance patient-provider dialogue [11], and in Sweden, a digital portal for young diabetes patients was found to facilitate trust and dialogue between patients and health care workers [13]. Moreover, a recent systematic review found that, more broadly, digitalization in health care is generally beneficial for improving communication-related factors like patient participation, treatment adherence, and self-management in chronic care [14]. Based on the Bertelsmann Digital Health Index, Germany is far behind most European neighbors with respect to digitization in health care, being placed second to last in terms of digital readiness, policy, and data use [15]. These shortcomings stem not only from slow implementation, but also from tools that fail to address real-world needs [16]. In this context, cocreation offers a pragmatic approach to developing fit-for-purpose tools by involving patients and other end-users directly in the design of digital health tools.

Digital services can help individuals navigate health care systems [17], book appointments [18], increase access to care [19], improve patient engagement [20], and enhance disease monitoring and management [21]. DHIs may be especially useful for migrants who often face challenges in navigating the health care system, making appointments, preparing for consultations, and engaging effectively with health care providers, in addition to language and cultural barriers [22]. International students form a key subgroup within the migrant population are central to Germany’s skilled migration strategy [23]. International students contribute to the economy through tuition fees, living expenses, and potential future participation in the skilled labor market if they remain in Germany after graduation [24]. Health care quality is a known factor influencing such decisions [25]. Therefore, understanding the needs of international students is not only critical to evaluating health care accessibility for future skilled migrants, but has broader economic implications for Germany [26].

The unique health care experiences and needs of the international student population in Germany have been largely unstudied, especially in primary care settings. However, some studies have been carried out exploring the health care experiences of young people in general. A cross-sectional study comparing university students in Germany and Belgium found that students in Germany were more likely to seek specialist and emergency care [27]. This pattern may indicate underusage of primary care services or difficulties navigating them. Moreover, there is evidence that individuals with a migrant background have higher odds of not having a general practitioner (GP), further suggesting that services may be underused [28]. These prior studies have highlighted communication barriers for broader migrant groups and refugees [29,30]. It is crucial to study international students as the intersection of these 2 populations to address the specific health priorities of this young, well-educated, and mobile population.

DHIs offer opportunities to personalize support and enhance communication before consultations. They also enable data capture on patient concerns, helping physicians develop more accurate patient profiles [31,32]. These tools are most effective when cocreated with end users to reflect their communication preferences, which often include clarity, empathy, and cultural sensitivity [33]. Behavioral science, particularly the Behavior Change Wheel (BCW) framework and the corresponding Capability, Opportunity, Motivation–Behavior (COM-B) model, offer a systematic way to map how digital tools can build patient capabilities, create opportunities, and motivate change [34]. The COM-B model conceptualizes behavior as arising from interactions between capability, opportunity, and motivation (COM), where capability refers to individuals’ knowledge and skills, opportunity to external factors that enable or constrain behavior, and motivation to reflective and automatic processes that drive action. It is embedded within the BCW, which provides a structured process for identifying target behaviors and selecting appropriate behavior change techniques (BCT). Previous studies have demonstrated that the BCW provides a systematic approach to identifying design considerations for behavior change interventions [35]. Cocreation helps align the design of tools with real-world considerations [36] and, when combined with the BCW, can help create acceptable and context-sensitive interventions for specific target groups [37].

To address the above challenges and opportunities, this study aims to adapt an existing digital patient preparedness tool (DiPPT) using cocreation with students at an international university in Germany, guided by behavior change theory. The cocreation workshops were structured using the PRODUCES framework [38], a cocreation framework for developing public health interventions that guides researchers through key stages of cocreation, including defining the Problem and Objective, planning the Design, identifying Users and Cocreators, and considering Evaluation and Scalability [38]. In the present study, the framework supported a systematic approach to end-user involvement, while the BCW [39] informed both the workshop topics and the derivation of intervention specifications from the workshop outputs. The workshops aimed to identify communication and preparation needs in primary care and to map behavior change levers for patient preparation using the COM-B model to inform the design of a digital intervention.

The study was guided by the following research questions which were investigated using different methods across a series of cocreation workshops:

1. How do international students prepare for conversations with physicians in German primary care settings?
2. What are the needs of international students with respect to receiving and relaying information in German primary care settings?
3. What are the waiting room experiences of international students prior to GP appointments?
4. What aspects of the preexisting DiPPT did international students see as suitable for a digital intervention to facilitate communication?
5. What aspects of the DiPPT did international students see as not suitable for a digital intervention to facilitate communication?
6. What are the needs of international students for a digital intervention to facilitate communication?

The methods used across the workshops demonstrate how cocreation can be used to identify end-user needs and translate these into design specifications for a behavior change intervention, which was a central objective of this Health CASCADE study.

## Methods

### Study Design

This study uses a convergent mixed methods design [40], with qualitative and quantitative data collected in parallel, to cocreate a digital health intervention for international students preparing for primary care consultations in Germany. The structure and methods of the cocreation workshops were specified in advance; building on a prior communication intervention (DiPPT) in obstetric care [41,42] and adapting it to the current target group.

Data integration occurred at the interpretation stage through narrative integration [43,44]. Quantitative survey data were used to descriptively characterize cocreators, summarize health care experiences, waiting room experiences, preparation behaviors, and evaluate ratings of existing DiPPT lessons. Qualitative data were used to explore and contextualize these experiences and behaviors in greater depth to identify needs and preferences for a redesigned digital intervention. See Table 1 for how qualitative and quantitative data were used to understand cocreators’ experiences and behaviors, and inform the adaptation of DiPPT for international students accessing primary care in Germany.

**Table 1.** Overview of cocreation workshops conducted with international students accessing primary care in Germany, aligned with stages of the BCW^a^ framework in a convergent mixed methods study.

BCW^a^ stage	Aim	Methods
Understand the behavior	Explore the communication experiences of international students accessing primary care.	Qualitative data Focus groups. Quantitative data Survey on doctor-patient communication.
Identify intervention options	Evaluate the relevance of content from an existing intervention (DiPPT)^b^.	Qualitative data Empathy maps exploring patient emotions and behaviors in waiting rooms. Quantitative data Survey on clinic waiting room experience. Rating each communication training component from DiPPT based on brief descriptions of each lesson.
Identify intervention options	Explore needs for a digital intervention in general as well as refine and prioritize content and features from the existing DiPPT for a redesigned digital tool.	Qualitative data Card sorting exercise of intervention ideas. Quantitative data Rerating each communication training component from DiPPT after having used the web-app.
Identify intervention options	Co-design and discuss the structure and core features of a tailored preparedness intervention.	Qualitative data Visioning exercises and cocreation of design charts. Focus groups to evaluate proposed intervention. Quantitative data Survey on demographics and health.

a BCW: Behavior change wheel.

b DiPPT: digital patient preparedness tool.

### Ethical Considerations

Ethical approval for the study was granted by the Ethics Committee at Constructor University (application number: 2022_01_B). All cocreators provided informed consent prior to taking part in each workshop and were informed about the purpose of the study, the types of data collected, and their rights as cocreators. Eleven out of 12 cocreators were employed as paid contributors and compensated equally based on the number of hours they contributed to the project (US $14.36/h). One cocreator was unable to receive payment due to visa-related employment restrictions and participated voluntarily. To protect cocreator privacy and confidentiality, audio recordings and workshop materials were anonymized with disclosure controls applied to names, health conditions, institutions, countries, and locations.

### Digital Patient Preparedness Tool

The original digital intervention (DiPPT) was developed to support communication between pregnant women and health care professionals in obstetric care [41,42,45,46]. The web-based tool delivered structured communication training through short lessons and exercises designed to help expectant mothers prepare for consultations and express concerns clearly. The intervention targeted safe communication behaviors during maternity care encounters and was informed by the Health Action Process Approach (HAPA) model, primarily using action planning (BCT 1.4) to encourage patients to plan for obstetric and midwife consultations ahead of time.

### Recruitment and Data Collection

To ensure the intervention was grounded in real-world experiences, international students were engaged as cocreators. The following section outlines how they were selected and involved in a series of cocreation workshops designed to explore patient behaviors and collaboratively adapt a digital preparedness tool using the BCW [39]. Additional details are mentioned in Supplement 1 in Multimedia Appendix 1 that consists of a full list of tools used to collect data from cocreators.

#### Selecting Cocreators

Cocreators were purposively sampled from an international university in Germany, using criterion sampling [47]. Eligibility criteria included being at least 18 years old, English-speaking, and having received primary care in Germany. Recruitment took place via in-class presentations, posters, flyers, and digital listings. Interested individuals completed an eligibility screening form and participated in a brief interview to gauge motivation for taking part. Twelve cocreators were selected. Of these, 10 attended all 4 workshops and 2 attended 3. Cocreators were relatively homogeneous with respect to demographic characteristics. All were between 19 and 22 years of age, and 9 of the 12 were female. Cocreators represented 6 academic disciplines, including psychology (n=5), chemistry and biotechnology (n=3), and computer science, economics, business administration, and medicinal chemistry (n=1 each). Eight cocreators were born in Asia or Africa, with the remaining cocreators originating from the Americas (ie, North, Central, South America) (n=2) and Europe (n=2) (Supplement 4 in Multimedia Appendix 2).

#### Cocreation Workshops

The aim of the cocreation workshops was to explore how international students in Germany prepare for health care consultations and to collaboratively adapt an existing digital communication intervention (DiPPT) into a preparedness tool tailored to this group’s needs. To design the intervention, the BCW [39] was used to inform the structure of the workshops: to first understand patient behaviors and then identify and refine potential intervention options. Workshop methods were sourced from consultation with experts in qualitative research methods and from the cocreation methods selector tool [48], which was developed within the Health CASCADE research network to support evidence-based cocreation in public health. To structure the cocreation workshops so that they could be evaluated in scientific terms, the PRODUCES framework was used [38]. The PRODUCES framework guides cocreation of public health interventions by structuring the planning, conduct, evaluation, and reporting of participatory processes, enabling a systematic and reproducible approach to intervention development [38]. Table 1 details how relevant stages of the BCW were operationalized in terms of workshop aims and methods used.

#### Quantitative Data Collection

A combination of nominal, Likert-scale, and free-text items was used in the survey instruments. All survey measures were developed specifically to explore patient needs and experiences in primary care, with a particular focus on communication and waiting room experiences. As there were no instruments with established psychometric properties that aligned precisely with the research focus, the bespoke tools were deemed fit for purpose. Furthermore, the need to capture target group–specific variables, minimize respondent burden, and reduce disclosure risk further supported the use of a short, tailored instrument rather than adapting existing scales designed for different contexts. Supplement 1 in Multimedia Appendix 1 consists of the full list of survey items.

#### Qualitative Data Collection

A variety of qualitative methods for data collection was used in the workshops to capture cocreators’ thoughts, opinions, experiences, and emotions both in primary care and concerning the requirements of a DHI designed to support international students in seeking health care. Focus groups were employed to elicit a broad range of experiences in primary care and to facilitate discussion of proposed interventions. These were supplemented by methods such as empathy maps to collect data on emotions and visioning exercises designed to stimulate creative thinking about adapting the original DHI to the primary care context.

### Data Processing and Analysis

#### Quantitative Analysis

Survey responses were processed using R (v4.5.1; R Foundation for Statistical Computing) [49] and RStudio (Posit PBC) [50], with health conditions categorized according to *International Statistical Classification of Diseases and Related Health Problems, Tenth Revision* (*ICD-10*) codes and countries of origin generalized to regions. Descriptive statistics and visualizations (bar charts, histograms, heatmaps) were used to summarize patterns in health status, waiting room experiences, and communication challenges; no inferential statistical analyses were conducted, as the quantitative data were solely intended to provide descriptive workshop-level insights alongside the qualitative findings. All code used to derive descriptives can be found in the Open Science Framework (OSF; Center for Open Science) repository, with the OSF Identifier: JBEAP [51].

#### Qualitative Analysis

Audio recordings from the focus groups were transcribed verbatim by a transcription agency. Transcripts were checked for accuracy by trained student assistants and anonymized using QualiAnon (v1.3.1; Qualiservice) [52]. Empathy maps, card sorting grids, and visioning charts were digitized using photographic records and recreated in JamBoard (Google LLC) for analysis.

Data was analyzed using structured qualitative content analysis (QCA) [53]. A coding frame was developed iteratively through deductive and inductive approaches. Initial categories were defined deductively based on the research questions and communication constructs from the original DiPPT, then expanded inductively through review of transcripts and workshop outputs. The final frame consisted of 12 parent categories, some with secondary layers, resulting in 52 unique codes. Supplement 2 in Multimedia Appendix 3 details the full coding frame.

Coding was carried out by 2 independent researchers (VAK and MS) across three iterative rounds using QualCoder (v3.5; Colin Curtain) [54]. Thematic segmentation was first applied to identify units of meaning. In each round, the 2 coders independently assigned codes to a shared subset of data (initial round: 45.95% of all segments), blinded to each other’s coding decisions during the initial coding stage. After each round, discrepancies were discussed and resolved either through consensus or by refining the coding frame. This two-pronged approach ensured both coder agreement and alignment with the data. As a result, the coding frame evolved in close collaboration between VAK and MS and was refined to improve consistency, clarity, and coverage. All frame iterations were documented to create an audit trail. Reliability and validity assessments were conducted after each round, yielding a final interrater agreement of 74.55% and a “miscellaneous” coding rate of 4.2%, indicating improved frame specificity and low residual ambiguity. Regarding the reliability assessment, code-specific metrics such as Cohen κ were not used due to the large coding frame relative to the sample set, which can distort agreement estimates when codes were infrequently used or unused [49]. Reliability and validity measures for each category are presented in Supplement 3 in Multimedia Appendix 4. Final coding was then completed by the lead researcher (VAK) using the validated frame. Outputs were extracted using custom SQL queries (accessed through OSF with the identifier: JBEAP) [51].

## Results

### Cocreators

Twelve international students participated in the cocreation workshops. Half reported living with a long-standing illness, including conditions related to mental health, musculoskeletal issues, and respiratory disorders. Despite this, most cocreators had visited a GP fewer than 5 times in the past year. Supplement 4 in Multimedia Appendix 2 provides detailed information about all data visualizations describing cocreators.

### Understanding Experiences and Behaviors of Cocreators

Cocreators described health care as an environment in which emotional uncertainty, system-level barriers, and communication breakdowns were common. Despite these challenges, many took active steps to prepare for appointments, suggesting that patient preparedness may be a promising target behavior. Insights from the workshops are presented below and summarized in Table 2. See Supplement 5 in Multimedia Appendix 5 for all data visualizations relating to cocreator experiences and behavior when receiving care.

**Table 2.** Topics, qualitative categories, example quotations, and most frequently used codes relating to health care experiences, waiting room experiences, and patient preparedness behaviors among international students in Germany.

Topic and category	Code	Example
Experiences of cocreators		
Assigning responsibility	Primarily doctor	“I agree in the sense that it’s both of their responsibilities, but I think it’s mostly the health care workers” [Cocreator 2, February 6, 2024]
Needs and complaints in primary care	Dignity in care	“The doctor is feeling maybe that I’m wasting his time, and it’s really not a nice feeling, like I’m being neglected” [Cocreator 5, February 6, 2024]
System challenges in Germany	Insufficient care	“Then, I feel like, okay, go on [REDACTED]’s page, look at the doctors that speak those languages, and then you look up their appointments and then they don’t have more appointments.” [Cocreator 4, February 6, 2024]
Emotions whilst waiting to see the doctor	Fear and anxiety	“The doctor came by and was like ‘Let me have a look at your hand.” He did some tests and then it was like ‘Okay, we’re going to get you to take an image.’ Then again, I was just waiting and I didn’t know whether I was supposed to tell somebody to take me to get the image or not. But then, after an hour and a half of waiting, someone came. However, there’s this creepy aura” [Cocreator 2, February 6, 2024]
Thoughts while waiting to see the doctor	Anticipation or expectation	“Am I having a heart attack?” [Empathy map – Chest pain, February 9, 2024]
Behavior of cocreators		
Preparation method	Taking notes	“Usually, I write down everything I have to say to the doctor” [Cocreator 3, February 6, 2024]
Preparation location	On the way	“Then maybe you revise it like when you’re on the train to refresh your mind” [Cocreator 11, February 6, 2024]
Preparation timing	Day before	“I think mostly like the day before” [Cocreator 11, February 6, 2024]
Reasons to prepare	Prompt the doctor	“I want to sometimes discuss with the doctor if this is a possible thing if this is a possible disease, or possible answer for whatever my symptoms are” [Cocreator 2, February 6, 2024]

Quantitative findings further showed that most cocreators viewed communication as a shared responsibility between physician and patient, although few felt that patients should take the lead role (Table 2 and Supplement 5 in Multimedia Appendix 5). Qualitative findings expanded on this pattern by demonstrating that cocreators generally expected physicians to assume primary responsibility for facilitating effective communication because of their duty of care or professional obligation.

#### Waiting Room Experiences

Quantitative and qualitative findings addressed different aspects of the waiting room experience. While survey data characterized where, with whom, and how long cocreators waited, qualitative findings highlighted the emotional experience of waiting. Survey data showed that the majority of cocreators arrived alone to appointments, had access to a waiting room, and waited inside the clinic. Waiting times varied considerably across cocreators; 6 waited less than 30 minutes on average, while 5 waited more than 30 minutes on average. The qualitative data showed that cocreators frequently experienced anxiety and uncertainty while waiting, often linked to anticipated diagnoses, unclear consultation expectations, or past experiences. This psychological vulnerability was one of the most frequently cited themes during empathy mapping activities.

#### Needs of Patients

##### Communication Needs and Challenges

**Figure 1.**
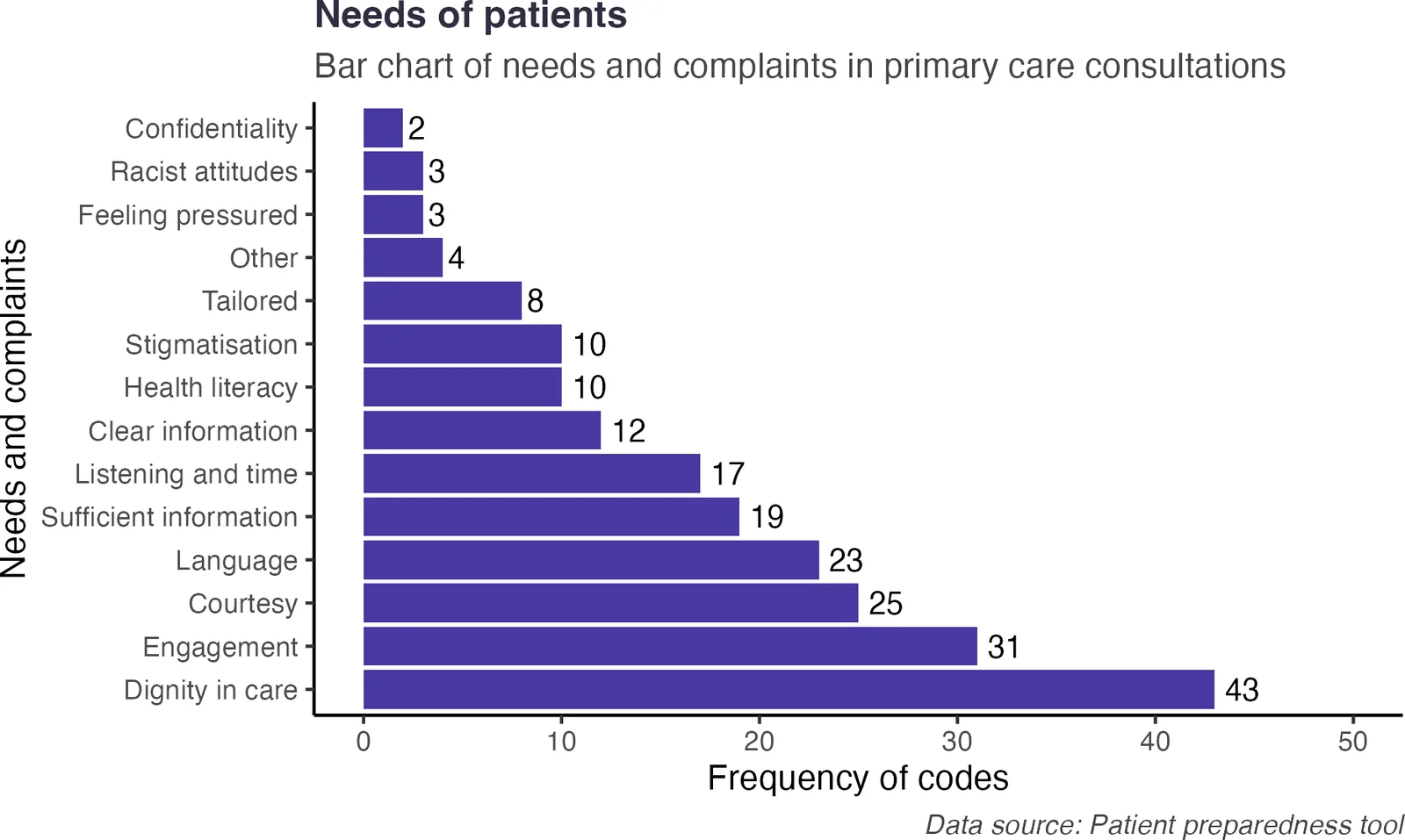
Frequencies of communication-related needs and complaints reported by international students during cocreation workshops on primary care experiences in Germany.

With respect to communication challenges that patients face, qualitative and quantitative data converge. Eight cocreators reported feeling misunderstood by health care workers in the quantitative data, and 4 reported not being listened to. Qualitative data expanded upon this by specifying the perceived causes of communication difficulties. Language barriers, lack of empathy and engagement, insufficient explanations, and dismissive behavior were frequently reported communication challenges across the focus groups. Figure 1 details the frequency of all codes used for the category “needs and complaints” in health care settings. Cocreators articulated a range of communication needs, including clearer explanations, culturally sensitive interactions, emotionally validating exchanges, and information delivered in plain language. Time constraints during consultations were also identified as a barrier to effective communication.

##### System Level Needs and Challenges

**Figure 2.**
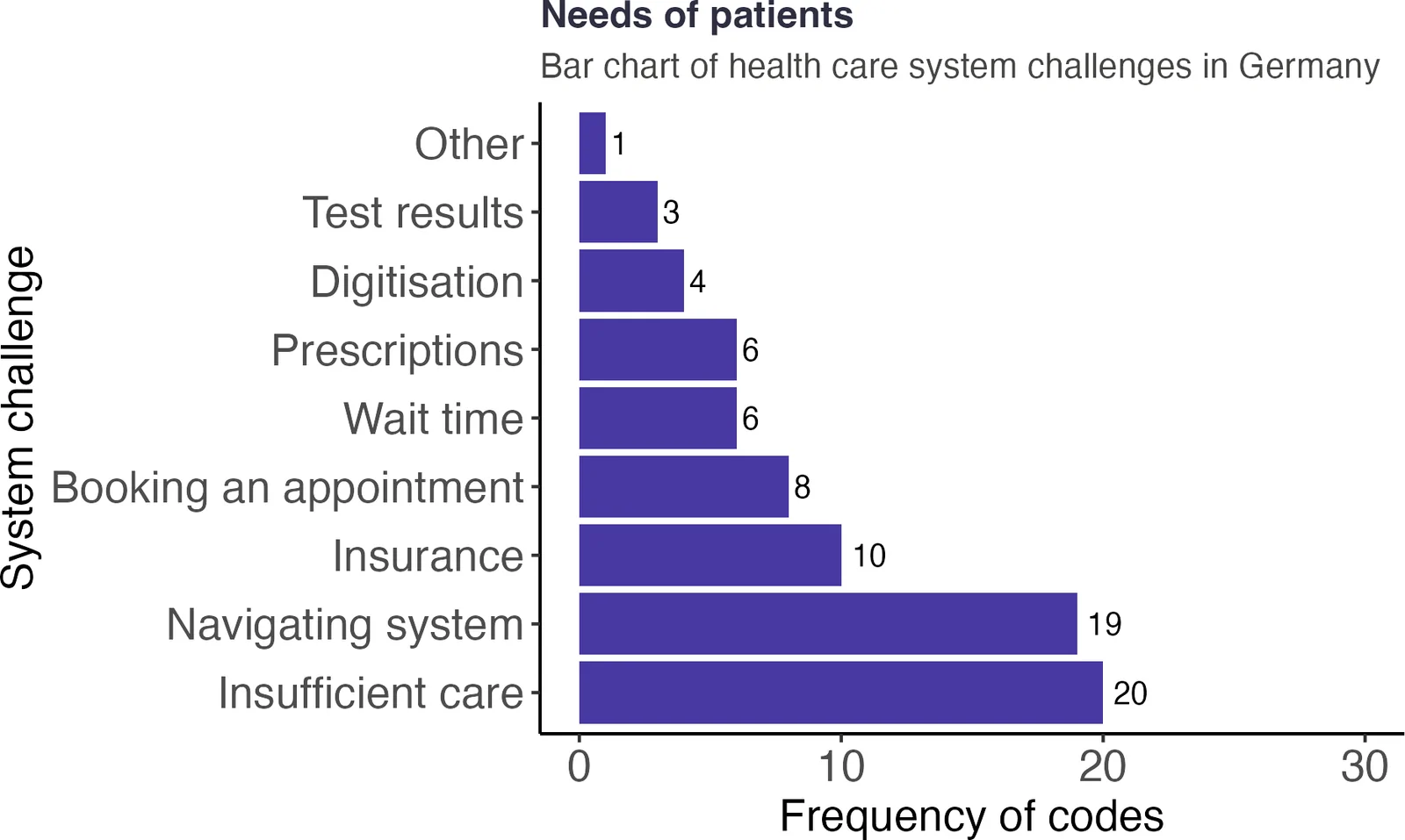
Frequencies of system-level challenges reported by international students when navigating the German health care system during cocreation workshops.

In addition to interpersonal issues, cocreators highlighted system-level needs in the focus groups such as improved access to appointments, better navigation support, and more user-friendly digital infrastructure, particularly for tasks like receiving test results. Complaints about difficulty navigating the system and inadequate digital services were among the most frequently cited challenges across the dataset. Figure 2 contains the frequency of all codes used for the category “system level challenges” in the German health care system.

### Patient Preparedness behavior

Focus groups revealed differences in how patients prepared for their appointments. Cocreators frequently described taking notes, listing symptoms, or researching conditions the day before or on the way to the clinic. These actions were intended to structure the consultation, ensure efficiency, and psychologically prepare for the encounter. “Taking notes” was one of the most frequently coded behaviors in the dataset. Some cocreators used preparation as a way to prompt the physician, express concerns clearly, or guide the direction of the conversation.

### Intervention Options

Qualitative data revealed that while the concept of a DiPPT was welcomed, the design must prioritize usability and relevance. Rather than structured training, cocreators sought lightweight, responsive features that could flexibly support them before and during primary care consults. These insights are detailed below and summarized in Table 3. Supplement 6 in Multimedia Appendix 6 outlines all data visualizations relating to cocreator needs for the digital health intervention.

**Table 3.** Qualitative categories, example quotations, and most frequently used codes relating to perceptions of the DiPPT^a^ and desired features for a redesigned digital preparedness intervention.

Category	Code	Example
Elements of DiPPT^a^ to retain	Retain content	“Maybe some lessons could be especially useful to a lot of people.” [Cocreator 5, February 20, 2024]
Elements of DiPPT to modify	Modify content	“Lesson 3 was frustrating, get to the point.” [Visioning flip chart – Lesson 3, February 20, 2024]
Features to include in the digital health tool	Communication training	"We had problems going to the Hausarzt explaining our state, like giving an explanation to the Hausarzt about our state, our symptoms. That was the main problem. If this app kind of helps in all of this, the communication between us and the doctors, then we can be better. " [Cocreator 10, February 20, 2024]
Features to exclude in the digital health tool	Open-ended responses	“When you’re typing a long answer... you expect something back.” [Cocreator 6, February 20, 2024]
Utility of tool	Limited use	“I don’t think I would use it multiple times...maybe just once if I have a really serious appointment.” [Cocreator 7, February 20, 2024]

a DiPPT: Digital Patient Preparedness Tool.

#### Suitability of the Existing DiPPT

**Figure 3.**
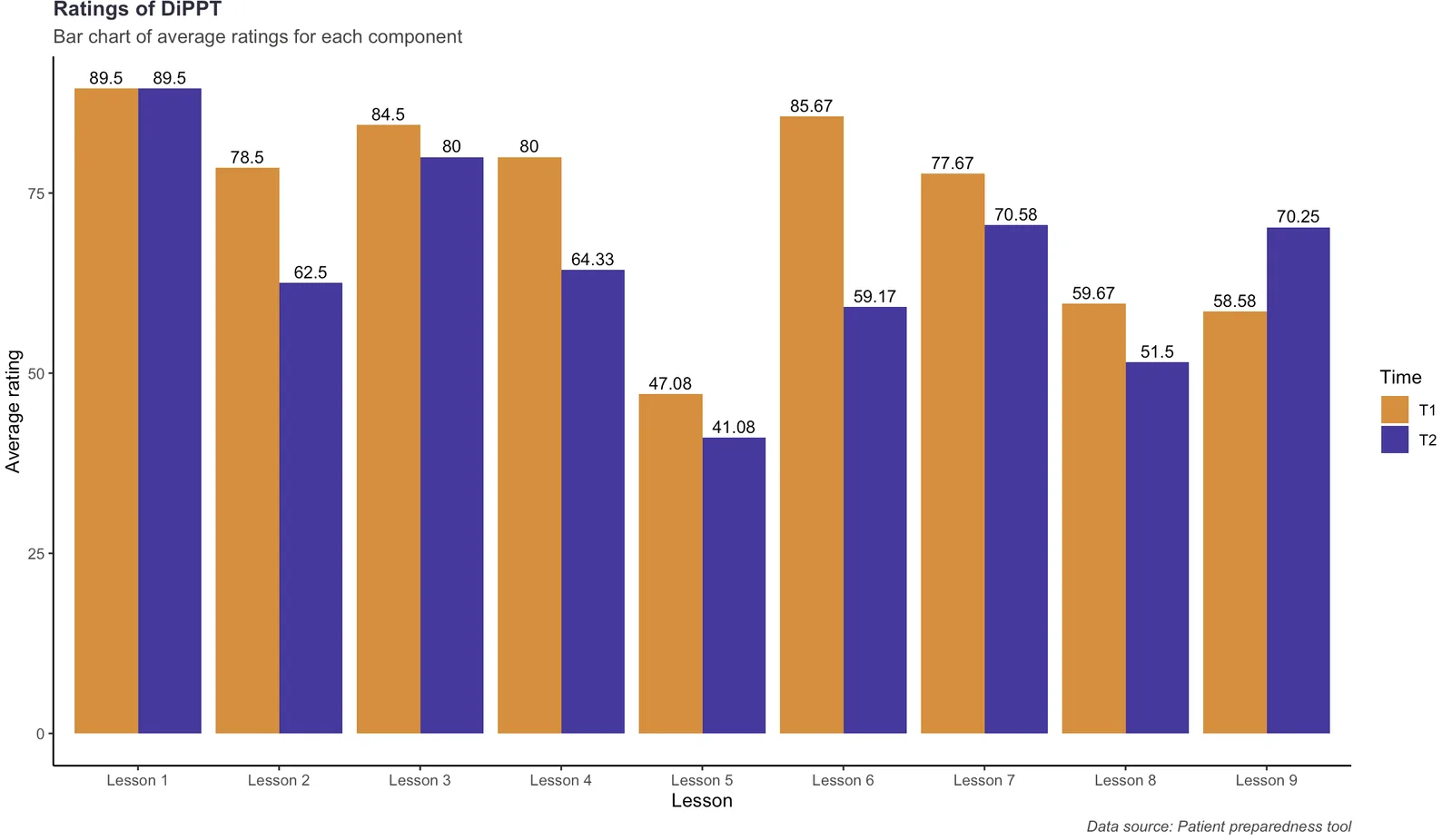
Average ratings of lessons from the digital patient preparedness tool (DiPPT) by international student cocreators before and after using the intervention during cocreation workshops in Germany.

Quantitative data identified the lessons that cocreators rated most and least favorably, while qualitative findings help explain both the content and delivery characteristics that may have contributed to these ratings. Figure 3 presents the average ratings cocreators assigned to each lesson before using the web-app (ie, with only a description of what was covered in the lesson) and after using the web-app.

With respect to intervention design, the data converge in suggesting a preference for concise, low-burden content. The highest-rated lessons were among the shortest in the intervention and contained relatively few open-ended or closed-ended exercises, while lesson 5, which had the lowest rating, contained the most open-ended activities of all lessons in the intervention. Qualitative data support this interpretation, with cocreators expressing dissatisfaction with lengthy lessons, quizzes, open-ended activities, and the existing lesson-based format.

With respect to communication support, the data converge in suggesting that cocreators valued content that helped them overcome barriers encountered during health care interactions, whereas lower-rated lessons asked patients to assume additional responsibility beyond overcoming those barriers. The highest-rated lessons focused on communicating information clearly (Lesson 1), speaking up about concerns (Lesson 3), and preparing for consultations (Lesson 7). Qualitative findings help explain this pattern, as cocreators frequently reported difficulties obtaining sufficient and clear information, having their concerns taken seriously, being listened to, and communicating effectively with health care workers. Lesson 3 directly addressed complaints relating to dignity in care, engagement, courtesy, and feeling heard by encouraging patients to express concerns and advocate for their needs. Lessons 1 and 7 addressed needs for sufficient and clear information by supporting patients in structuring information, asking questions, and seeking clarification. Lesson 1 additionally provided strategies for communicating clearly, which may be particularly relevant for cocreators who reported language-related communication difficulties.

The data also converge in suggesting that content perceived as shifting responsibility towards patients was viewed less favorably. Two of the lowest-rated lessons focused on empathizing with the doctor (Lesson 5) and structuring information for the doctor (Lesson 6), both of which emphasized adapting patient behavior to facilitate communication with health care workers. Qualitative findings support this interpretation, as most cocreators expected physicians to take primary responsibility for communication because of their professional role and expertise.

The data further suggest that alignment with cocreators’ existing behaviors and perceived needs influenced lesson ratings. Lesson 7, which received a relatively high rating, aligned closely with preparedness behaviors identified in the workshops, including taking notes, prioritizing concerns, and preparing questions before consultations. In contrast, Lesson 8, which received a relatively low rating, did not directly address the communication difficulties or health care navigation challenges raised by cocreators. Instead, it focused primarily on stress management and may therefore have been perceived as less relevant to their immediate needs.

Together, the data suggest that cocreators valued practical communication support that directly addressed challenges encountered during primary care consultations, while content perceived as burdensome, less relevant, or shifting responsibility towards patients was viewed less favorably.

#### Needs for a Digital Tool

Quantitative lesson ratings indicated general acceptance of communication-related content, while qualitative data identified communication support as the most frequently requested feature. Together, the data suggest that communication support may be a useful component of a digital preparedness intervention for international students.

**Figure 4.**
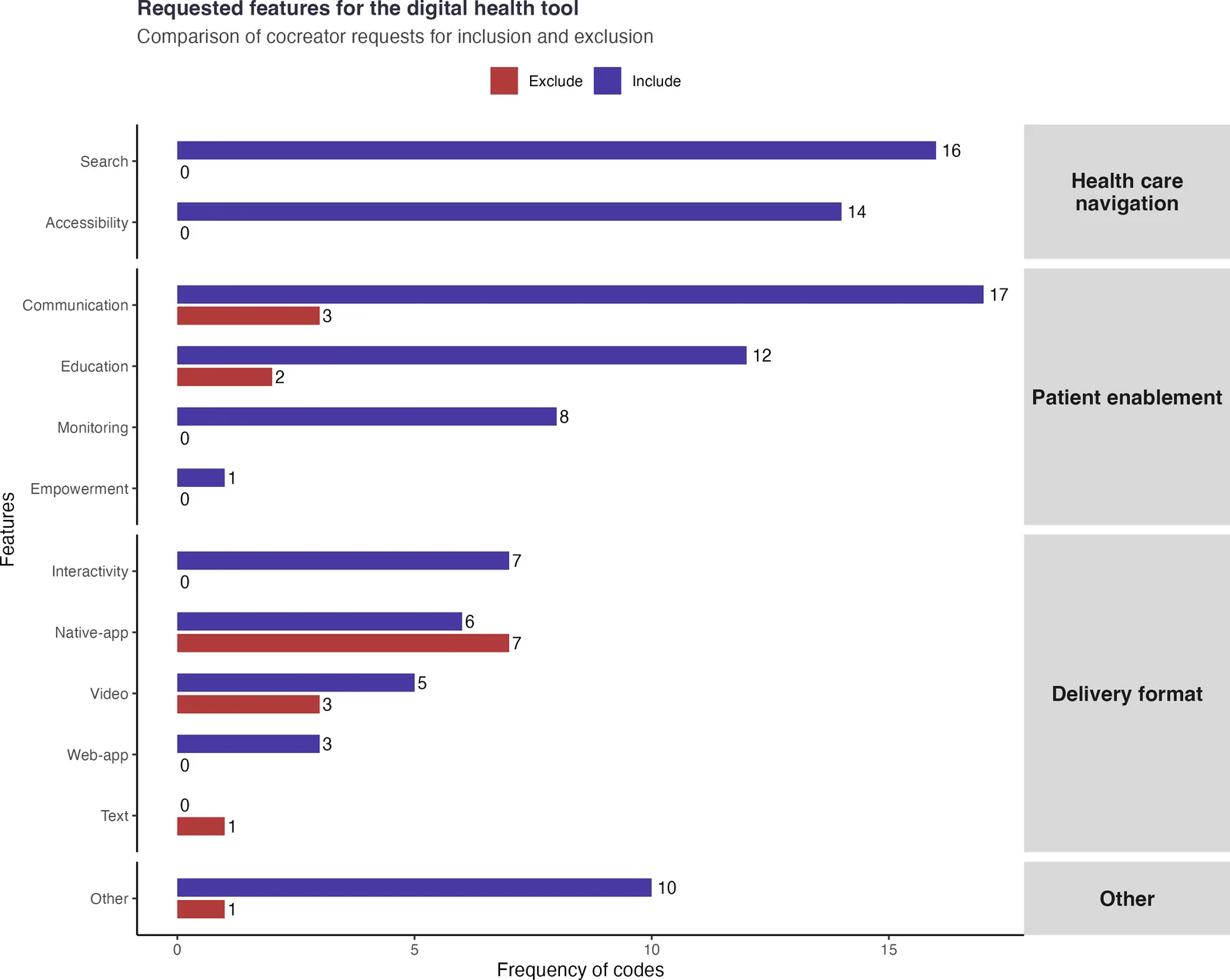
Frequencies of features requested for inclusion and exclusion in a redesigned digital health intervention (DHI) for international students accessing primary care in Germany.

Qualitative data from focus groups, card sorting activities, and visioning exercises identified several additional functional needs. These included support for appointment scheduling, clinic search functions, access to patient rights and symptom information, health monitoring tools, personalized feedback, and interactive features such as chatbots. The most frequently requested features were communication prompts, scheduling functions, and interactive components. Figure 4 includes the features cocreators requested to be included and excluded from the design specifications for a digital health intervention.

Qualitative data also highlighted reservations regarding adoption of the intervention. Many cocreators indicated that they would use such a tool only under certain conditions or not at all, particularly if it addressed only doctor-patient communication. Several cocreators suggested that the perceived value of the intervention would increase if it also supported broader health care needs, such as appointment scheduling, clinic navigation, or access to health care information.

Quantitative and qualitative data provided complementary insights into the suitability of delivering the intervention through a digital medium. While survey data indicated that most cocreators could use mobile devices while waiting for appointments, limited Wi-Fi availability and variable mobile data quality may restrict digital engagement in practice. Qualitative findings further highlighted concerns regarding the usefulness of a standalone communication app. Together, these findings suggest that successful implementation may depend on both the digital environment in which the intervention is used and the extent to which it addresses needs beyond communication support.

## Discussion

### Principal Findings

This study adopted a convergent mixed methods design to cocreate a DiPPT with students from an international university in Germany. To our knowledge, this study is the first to explore the health care needs and experiences of international students accessing primary care in Germany and to use these insights to cocreate a DiPPT. Quantitative and qualitative data together captured international students’ health care experiences, preparation behaviors, and perceptions of a digital preparedness intervention. Integrating quantitative and qualitative findings enabled interpretation of both the prevalence and context of cocreators’ experiences. Survey findings provided descriptive insight into communication perceptions, health care experiences, and waiting room conditions, while qualitative findings contextualized these patterns by exploring emotional vulnerability, preparation behaviors, and intervention preferences in greater depth. Cocreators’ needs explored in the qualitative data included both interpersonal communication needs and challenges as well as broader system-level barriers related to navigating the health care system and accessing services. Importantly, these findings suggest that the health care experiences of international students may reflect the more general experience of young adults navigating an unfamiliar health care system independently for the first time. Collectively, the data informed a design specification for a digital intervention tailored to international students in Germany while demonstrating a replicable approach for integrating cocreation methods and the BCW in digital health intervention development.

In the survey data, most cocreators agreed that communication should be a shared responsibility between physician and patient, and the majority disagreed that patients should carry the lead role. Qualitative findings indicated that despite the shared responsibility, physicians were expected to take the lead due to professional responsibility. The expectation that both parties should contribute to communication is consistent with evidence that younger people increasingly wish to participate actively in health care decisions rather than adopt a purely passive role [55,56]. Similarly, recent work on Generation Z has found that young adults value collaborative relationships with health care providers, leveraging digital fluency to become more health literate and actively taking part in decision-making [57]. However, expectations regarding who should lead the consultation may also reflect previous health care experiences in cocreators’ countries of origin. Most cocreators in the present study originated from Asia and Africa, where shared decision-making often differs from the Western European and North American model. Across many South Asian settings, patients commonly value involvement while expecting physicians to provide recommendations and assume greater responsibility for directing consultations [58-60]. Evidence from African settings is more heterogeneous, but similarly suggests that clinician authority remains influential in many contexts, while patients continue to value involvement in decisions [61,62]. Taken together, these findings suggest that the pattern observed in this study may reflect the combined influence of being a young adult seeking active involvement in health care while drawing on previous experiences of health care systems in which physicians are expected to take greater responsibility for guiding clinical discussions.

Findings from the survey data further showed how many cocreators felt misunderstood or not listened to, while qualitative findings provided insight into the perceived causes of these experiences, including language barriers, insufficient explanations, lack of empathy, and dismissive behavior. Across the focus groups, cocreators reported feeling emotionally vulnerable during primary care interactions. For international students, these experiences may be compounded by the transition to a new health care environment.

Cocreators described not only communication difficulties during consultations but also uncertainty about how health care services operate in Germany. This suggests that experiences of care are influenced by both being recent migrants and navigating an unfamiliar health care system independently for the first time. Emerging adulthood is characterized by increasing responsibility for managing one’s own health care, including recognizing when to seek care, arranging appointments, selecting providers, communicating symptoms, and making health care decisions without parental support [63]. Previous research has shown that young adults commonly experience uncertainty about how health care systems operate, question whether their symptoms warrant seeking care, worry about whether their concerns will be taken seriously, and often lack the health care knowledge and confidence developed through prior experience [64,65]. These challenges may be further amplified when young adults are simultaneously adapting to a new health care system with unfamiliar organizational structures, administrative processes, and communication norms. Previous studies in Hungary, Turkey, and the United States have reported similar findings: international students experienced language barriers, difficulties with appointment and insurance processes, perceived lack of dignity or insufficient care, and challenges navigating health care services [66-68]. These findings echo broader research documenting negative health care experiences among migrant groups in Germany, particularly concerning unclear communication, perceived indifference, cultural barriers and lack of dignity in care [69-72]. Complaints of dismissiveness and language barriers were among the most frequently coded responses, aligning with broader literature on migrant health care experiences in Germany [73,74].

This study confirms that in addition to provider demeanor, system-level needs and challenges also shape health care experiences of international students. While the surveys primarily characterized communication experiences and waiting room conditions, qualitative findings additionally identified broader system-level challenges, including difficulties arranging appointments, identifying suitable providers, and accessing digital infrastructure. These findings align with recent research showing that migrant groups in Germany often face structural barriers when navigating health care systems, including limited information about available services and administrative complexity [22]. While these challenges are common among migrant populations, they may have particular implications for international students, who must navigate unfamiliar health care processes independently for the first time. These findings highlight the importance of accessible, efficient, and supportive health care encounters for individuals who are unfamiliar with the health care system. Studies of digital health systems also suggest that insufficient integration of digital services and patient-facing tools within existing health care infrastructures can further limit accessibility and usability [14,75].

Cocreating an intervention with international students provided insight into how health care support may need to be tailored to this population. Cocreators preferred concise, practical, and multifunctional digital tools that could support both consultation preparation and broader health care needs.

Qualitative data also indicated that most cocreators actively prepared for consultations by taking notes and researching symptoms, with a motivation to improve communication efficiency and manage emotional stress. Preparation strategies were implemented just prior to appointments and were often used to prompt physicians during the consultation. This mirrors findings from previous studies highlighting that patients who prepare in advance are more likely to participate actively in clinical conversations [45,76]. While cocreators accepted shared responsibility for communication, they generally expected more engagement and empathy from providers, reinforcing the need for bidirectional communication efforts [77,78].

Feedback on the current DiPPT was mixed. Integrated qualitative and quantitative data suggested that perceptions of the intervention were influenced by both the content and delivery format of the lessons. The highest-rated lessons addressed challenges frequently identified by cocreators throughout the workshops, including obtaining clear information, communicating concerns, and preparing for consultations. In contrast, lower-rated lessons were perceived as more burdensome, less relevant to cocreators’ immediate needs, or as placing additional responsibility on patients rather than health care providers. This latter finding converges with cocreators’ views regarding communication responsibility, with cocreators generally expecting physicians to take the lead role in facilitating effective health care communication. Qualitative data further indicated dissatisfaction with lengthy lessons, quizzes, and open-ended exercises, while quantitative findings showed that the highest-rated lessons were among the shortest and least demanding components of the intervention.

Together, the data suggest that intervention acceptability may depend not only on addressing communication challenges experienced by patients, but also on presenting support in a concise, practical, and low-burden format that aligns with cocreators’ expectations of the doctor–patient relationship. Similar to findings in the literature [11,45], digital interventions that incorporate personalized feedback and patient-centered support are more likely to be adopted. However, many cocreators expressed skepticism about routine tool use, emphasizing that added features were necessary to drive engagement [79,80].

### Implications

The BCW was used to derive design specifications for a DiPPT tailored to international students [81]. Cocreation insights were mapped onto COM-B determinants (Capability, Opportunity, and Motivation) to identify key behavioral drivers of patient preparedness (the target behavior) among international students (the target audience). Capability-related needs included structured communication support (eg, checklists), opportunity-related factors reflected physician encouragement and system integration, and motivation-related factors emphasized interactive and multifunctional features (eg, chatbots). The relationship between these determinants and resulting intervention features is summarized in Supplement 7 in Multimedia Appendix 7.

As a potential implementation pathway, the intervention may be embedded into university orientation programs and distributed via clinic-based pamphlets, thereby normalizing the practice of patient preparation among students. Moreover, orientation programs may benefit from providing international students with clearer information about how the German health care system functions. This approach is echoed by Abdulai and colleagues [82], who emphasize the importance of considering contextual factors and leveraging existing university support structures when supporting international students. Informing students about available health care services, as well as existing tools and resources for finding clinics, booking appointments, and accessing digital health services, may help alleviate some of the navigation challenges reported by cocreators in the workshops. Taken together, these findings offer a behaviorally grounded and contextually responsive foundation for intervention development.

This study illustrates the value of combining behavioral science frameworks such as the BCW and COM-B model with systematic cocreation methodologies, including the Health CASCADE guidelines [83] and the PRODUCES framework [38]. The PRODUCES framework helped in conceptualizing the problem, communicating the objective and design of, as well as identifying the cocreators to recruit as part of the workshops, and in determining the scaling strategy to use. The BCW played a key role in shaping the sequence and focus of the workshops, facilitating the identification of end-user needs and behavior change determinants. The design of the workshops was also guided by the work of Health CASCADE, who have developed cocreation guidelines [83], to create an inclusive and transparent process and ensure that cocreators’ voices were meaningfully integrated into the intervention’s design. By combining these methodologies with the BCW and COM-B, the study provides a replicable and scalable roadmap for developing digital health interventions that are grounded in theory and shaped by the lived experiences of users by means of acting as a role model. Together, these frameworks support the creation of interventions that are sensitive to real-world user needs, anchored in validated behavior change methods, and designed with sustainability in mind through participatory development practices.

### Feasibility

While the previous section outlines the behavioral determinants and design specifications of the intervention, questions remain regarding its feasibility and potential adoption in real-world settings. Although cocreators expressed preferences for an intervention that would support doctor-patient communication, several indicated that they would likely use such a tool only once or before particularly important medical appointments.

The data also highlighted challenges relating to the digital medium. Together, the limited connectivity and skepticism towards a standalone communication app suggest that the intervention may function primarily as a situational preparation aid rather than a tool intended for continuous engagement. This pattern aligns with broader findings in digital health research showing that initial interest in digital tools often does not necessarily translate into sustained use [84,85]. Many digital health interventions experience rapid declines in engagement following early exposure, even when users report positive perceptions of their usefulness [86,87].

Adoption of digital health tools is also influenced by the broader environment in which they are deployed. Cocreators highlighted that the perceived value of a preparedness tool would increase if it addressed system-level challenges, such as appointment scheduling, clinic navigation, or access to medical documentation. Prior research has shown that digital health interventions are more likely to be adopted when integrated into existing care infrastructures rather than functioning as standalone behavior change tools [88,89].

If the intervention is primarily used as a one-off preparation tool, its effectiveness may therefore depend on where it is embedded within existing systems, such as appointment booking platforms, university health services, or patient portals. Future work should therefore explore which digital tools and communication channels international students currently rely on when navigating health services in Germany, as this may reveal more feasible points of integration. In some contexts, a lightweight intervention embedded within existing platforms or informational resources may be more appropriate than a standalone application.

### Limitations

To enhance cocreation and better understand the mutual communication between physicians and patients, future studies should consider involving physicians and clinic administrators as cocreators in the process. Additionally, the coding frame used for qualitative content analysis was limited in distinguishing between specific content, design, and functional preferences for digital tools. Future iterations should further refine coding categories with respect to specific aspects of the DiPPT that should be retained or excluded, and further delineating the miscellaneous categories for cocreator needs of a digital health intervention.

Furthermore, quantitative data were collected anonymously, preventing direct linkage with qualitative insights by person. Future designs should link cocreator data to deepen the understanding of tool acceptability among distinct user profiles. Finally, cocreation prioritizes depth of insight over statistical representativeness, so the study involved a small sample of international students (n=12), homogeneous with respect to age (19‐22 y), enrolled at a single English-speaking international university in Germany, limiting generalizability. Therefore, the findings presented here should not be interpreted as reflecting the needs of the broader migrant population in Germany. Moreover, the extent to which the findings are specific to international students, reflect broader migrant experiences, or arise from the intersection of student and migrant identities remains unclear. Further research is needed to disentangle these influences and examine the transferability of the findings to other populations. However, the methodological approach may be transferable to other contexts, and future research should extend this approach to diverse academic institutions, age groups, and include a wider range of cultural backgrounds.

The detailed reporting of the cocreation procedures and the transparent presentation of the coding frame provide a foundation that future researchers can use to inform the design of workshops with other migrant subgroups and settings.

### Conclusion

In conclusion, this study identified key communication and system navigation challenges faced by international students in German primary care and translated these into design specifications for a tailored DiPPT. By integrating cocreation methods with the BCW and COM-B model, the study offers a foundation for future intervention development.

## Supplementary material

10.2196/87519 Multimedia Appendix 1 Tools used to collect data from cocreators.

10.2196/87519 Multimedia Appendix 2 Visual summaries of cocreator demographics and health status.

10.2196/87519 Multimedia Appendix 3 Final coding frame used for qualitative content analysis.

10.2196/87519 Multimedia Appendix 4 Interrater agreement and coding frame validity metrics.

10.2196/87519 Multimedia Appendix 5 Visual summaries of health care experiences and preparation behaviors.

10.2196/87519 Multimedia Appendix 6 Visual summaries of needs and preferences for digital health tool design.

10.2196/87519 Multimedia Appendix 7 Detailed specifications for a health behavior change intervention for international students in Germany based on the cocreation workshops.

## Abbreviations

BCT: behavior change technique
BCW: behavior change wheel
COM-B: Capability, Opportunity, Motivation – Behavior
DHI: digital health intervention
DiPPT: Digital Patient Preparedness Tool
GP: general practitioner
HAPA: health action process approach
*ICD-10*: *International Statistical Classification of Diseases and Related Health Problems, Tenth Revision*
OECD: Organization for Economic Cooperation and Development
OSF: Open Science Framework
QCA: qualitative content analysis
