# Supplementary material for: Cocreating a Digital Patient Preparedness Tool for International Students Accessing Primary Care in Germany: A Convergent Methods Study Within the Health CASCADE Network
Source: JMIR Hum Factors. 2026 Sep 14;13:e87519. doi: 10.2196/87519 (PMC13574332; doi:10.2196/87519)
Supplement: Supplementary file 1 [file humanfactors-v13-e87519-s001.docx]

# Data collection tools

Table 1. Table of variables collected in survey instruments and operationalisation of each.

| Variable | Operationalisation | | |
| --- | --- | --- | --- |
|  | Item wording | Item type | Response options |
| Age | Age: | Free text |  |
| Sex | Sex: | Free text |  |
| Major | Major (please write out in full i.e. no acronyms) | Free text |  |
| Language | Languages you speak | Free text |  |
| Language proficiency | The proficiency of each (e.g. English C1, German A1 etc) | Free text |  |
| Countries resided in | Please list the countries you have resided in | Free text |  |
| Countries received healthcare in | Please list the countries you have received health care services in | Free text |  |
| Country of birth | Country of birth: | Free text |  |
| Year of arrival in Germany | If you were born overseas, please provide year of arrival in Germany: | Free text |  |
| Pre-existing health conditions status | Do you currently have any pre-existing medical conditions or chronic diseases? | Free text |  |
| Pre-existing health conditions list | Please list all that apply: | Free text |  |
| Visits to the doctor | Please note down how often you visited the Haus Arzt/ your GP in the past year: | Free text |  |
| Responsibility attributed to healthcare workers, patients and both with respect to communication in a healthcare setting | Whose responsibility is it to ensure that communication works well in a healthcare setting? (please rate the extent to which you agree with each response)  Healthcare worker only  Patient only  Both | Likert Scale | 5: Strongly agree \|  4: Agree \|  3: Agree nor  Disagree \|  2: Disagree \|  1: Strongly disagree |
| Feeling misunderstood in a healthcare setting | When working with healthcare workers, has it ever happened to you that you felt misunderstood? | Nominal | Yes \| No \| Not sure |
| Examples of feeling misunderstood by healthcare worker | If you answered yes to the previous question, can you please provide an example? | Free text |  |
| Not feeling listened to by healthcare worker | When working with healthcare workers, did you typically feel that the healthcare worker listened to you? | Nominal | Yes \| No \| Not sure |
| Method for arranging doctor’s visit | How do you arrange your doctor’s visit? | Nominal | Call ahead and making an appointment \| Walking into the clinic and waiting \| Other (please state) |
| Whether accompanied for doctor’s visit | Do you typically go by yourself to the clinic? | Nominal | Yes \| No \| Other (please state) |
| Interactions during doctor’s visit | Who do you interact with when you visit the GP office? (please select all that apply) | Nominal | Doctor \| Receptionist \| Nurse; Patients  \| Other (please state) |
| Crowd inside clinic | How busy is the clinic on average? | Ordinal | Very busy \| Somewhat busy \| Not busy |
| Waiting time in clinic | How long do you typically wait? (please write down in minutes) | Free text |  |
| Location where patients wait | Where do you wait? | Nominal | Inside the clinic \| Outside the clinic \| Other (please state) |
| Waiting room in clinic | Does your clinic have a waiting room? | Nominal | Yes \| No \| Other (please state) |
| Seat in waiting room | If your clinic has a waiting room, do you usually get a seat in the waiting room? | Nominal | Yes \| No \| Other (please state) |
| Mobile devices in clinic | Are you allowed to use your mobile device in the waiting room? | Nominal | Yes \| No |
| Wifi in clinic | If you are allowed to use your phone, does your clinic have wifi? | Nominal | Yes \| No \| Other (please state) |
| Mobile data in clinic | If you are allowed to use your phone, what is the quality of your clinic’s mobile data connection? | Ordinal | Weak \| Moderate \| Strong \| Other (please state) |

Table 2. Table presenting the topic guide for each focus group.

| Workshop | Focus group topic guide |
| --- | --- |
| 1 | We will start by looking at the results from the short survey you just filled out and then run through some more open ended items.  Let’s take a look at how the group responded to the item about responsibility.  Would anyone like to share their response to this item, the reason behind their response, and what role each party could play in communication?  Would anyone like to share a situation when they felt misunderstood or not listened to when interacting with a healthcare worker?  What are the challenges of providing information to health care workers?    What are the challenges of receiving information from health care workers?    Do you generally feel like you need to prepare for your doctor’s appointments? What do you need to help prepare?    If you would like to prepare:   - where would you generally like to prepare for your appointment? (e.g. at home, on the bus, in the clinic etc) - when would be an ideal time to prepare? (e.g. day before, on the day etc)     Thinking back to your last doctor’s appointment, did you prepare for the session? If yes, can you share how you prepared?    Is there anything you would do differently the next time you see the doctor?    You’ve mentioned that you do the following […] to hep prepare for a doctor’s appointment. In the field of doctor-patient communication, the following factors are considered important:   - Providing sufficient information - Providing accurate information - Providing information clearly - Providing information that is relevant to the current context - Providing information in a way that is suited to the individual   Let’s go through each of them and see if these are things you try to do too.  Before we wrap up for the day, would anyone like to share anything else relating to their experiences with healthcare workers? |
| 4 | We’d like to do a round table with a series of questions:   - Would you use the tool we are developing? (i.e. something that is adapted from DHI A for patient preparedness) - If you were in charge of developing this tool, what would be your next steps?   If we did build this tool, how would you evaluate it? (i.e. how would you test that it is effective/ or is doing what we set out to do)  Taking a step back, what would be the best way of helping people prepare for their appointments? (e.g. an app, online videos, workshops etc)  Before we do our last activity together, would anyone like to share anything else relating to this tool? |

Table 3. Table with instructions for the interactive activities in workshops.

| Activity | Instructions |
| --- | --- |
| Empathy maps | 1. In groups, please build an empathy map of someone waiting to see the GP. 2. The case each group is provided is there to see the doctor for a different reason:    a. One case is there to get a sick note    b. One case is there to help treat a persistent cough    c. One case is there to enquire about chest pains and shortness of breath 3. A template is provided to fill out. Groups are encouraged to draw and name the case. Please discuss as a group, what the person might say feel, think and do whilst waiting to see the doctor. |
| Visioning | 1. In groups, please adapt lessons from DHI A so that it can help patients prepare for primary care consultations. Please think about how you would change the lesson to help someone speak to their doctor. 2. Each group is assigned one of the following lessons:    a. Lesson 1    b. Lesson 3    c. Lesson 7 3. In you groups, please use the iPad to go through the lessons and note down what you like and don’t like about the lesson 4. After reviewing the lesson as a group, do a round table to share observations about the lesson 5. Using the flipchart, post-it notes and markers, please brainstorm how would adapt this lesson for someone about to speak to their doctor (i.e. how would you change it?). Here you can use a mindmap, sketch your interface, draw pictures – whatever helps generate ideas from the group. Please address the following in the brainstorm to adapt the lesson into a patient preparedness tool: (10 min)    a. How would you structure the lesson?    b. How would you present the content from the lesson?    c. What other changes would you introduce to make the lesson more useful, effective or appealing in a patient preparedness tool? 6. Create a two minute pitch of your ideas to adapt the lesson into a patient preparedness tool. You can use your flipchart in the pitch if you feel this will help. Please ensure you cover the following bits of information in your pitch (4 min)    a. How does your lesson add value to the patient preparedness tool?    b. What have you kept from DHI A?    c. What have you dropped/ changed from DHI A?    d. Provide a summary of how you’ve adapted it to a general practice setting? |
