# Supplementary material for: Cocreating a Digital Patient Preparedness Tool for International Students Accessing Primary Care in Germany: A Convergent Methods Study Within the Health CASCADE Network
Source: JMIR Hum Factors. 2026 Sep 14;13:e87519. doi: 10.2196/87519 (PMC13574332; doi:10.2196/87519)
Supplement: Supplementary file 2 [file humanfactors-v13-e87519-s002.docx]

# Co-creator descriptives

Figure 1. Bar chart of sex distribution of co-creators.

Figure 2. Histogram of age distribution of co-creators.

Figure 3. Bar chart of educational background of co-creators.

Figure 4. Stacked bar chart of region of birth and regions where co-creators have received healthcare services.

Figure 5. Histogram of time since arrival in Germany.

Figure 6. Scatterplot of regions resided in by each co-creator prior to arriving in Germany.

Figure 7. Heatmap of underlying health condigions and number of visits over the past 12 months to the primary care physician.

Figure 8. Bar chart of long-term or underlying diagnoses reported amongst co-creators
