# Supplementary material for: Cocreating a Digital Patient Preparedness Tool for International Students Accessing Primary Care in Germany: A Convergent Methods Study Within the Health CASCADE Network
Source: JMIR Hum Factors. 2026 Sep 14;13:e87519. doi: 10.2196/87519 (PMC13574332; doi:10.2196/87519)
Supplement: Supplementary file 3 [file humanfactors-v13-e87519-s003.docx]

# Coding frame

Table 1. Table of codes form the level one parent categories and definitions of each.

| L1 Category | Code | Definition | Example |
| --- | --- | --- | --- |
| Needs and complaints | Sufficient information [deductive] | This sub-category is to be used if a co-creator states that they expect health care workers to provide comprehensive, or relevant information relating to their health concern or query. It is also to be used when co-creators complain that healthcare workers currently do not provide comprehensive or relevant information. | “After that, after the whole visit, when it comes to feedback, it's really poor.” |
|  | Clear information  [deductive] | This sub-category is to be used if a co-creator states that they expect their health care worker to express messages clearly, precisely, in plain English or avoid jargon. It is also to be used when co-creators complain that healthcare workers currently do not provide clear, precise or jargon free information. | “I had a problem going back and forth every day, waking up early so that there wouldn't be people there, and they didn't give me the exact information of where I needed to go or what I needed to do.” |
|  | Language  [inductive] | This sub-category is to be used if a co-creator complains that the healthcare worker does not speak or does not make an effort to interact with them in English. It is also to be used when co-creators express a wish for healthcare workers to speak more English. | “The first time i went to the doctor here in Germany I was looking for a Hausarzt. Language was definitely a boundary so I feel like there was a lot of confusion and misunderstanding during the session” |
|  | Health literacy  [inductive] | This sub-category is to be used if a co-creator expresses a wish to have greater health literacy to facilitate their interaction with healthcare workers. It is also to be used when co-creators complain that they don’t have sufficient knowledge to converse with healthcare workers or they have challenges in reconciling or making sense of symptoms and their own health. | “Then I feel like I'll be going over my online research results because I don't have the medical experience that they do. Obviously, you can’t just get all the information online, but sometimes it a thing.” |
|  | Courtesy  [inductive] | This sub-category is to be used if a co-creator complains that the healthcare workers are rude, not polite or interrupted them. It is also to be used when co-creators express a wish that their healthcare workers would be more courteous. | “The lady that welcomed  me at the info part was extremely rude.” |
|  | Dignity in care  [inductive] | This sub-category is to be used if a co-creator complains that they do not feel acknowledged, seen, respected or treated as an equal by healthcare workers. It extends to feeling that their symptoms are not being taken seriously, and a wish by co-creators to have validation and acknowledgement from healthcare workers. This can also be expressed as a wish for a safe environment or one that is less intimidating whilst receiving care. It is also to be used when co-creators request for a safe space or better bedside manner from the doctor. | “Said I over exaggerated my pain only to find out later that I did not” |
|  | Engagement and inquiry  [inductive] | This sub-category is to be used if a co-creator complains that healthcare workers do not ask enough questions or engage with them during a medical consult. Engagement refers more broadly to interactions with patients through conversation. It is also to be used when co-creators express a wish that the healthcare worker would ask more questions or talk to them more. | “In my case, I've just been to the general practitioner,  and they just ask you one question like, "Is your stomach paining?" Then I'm like, "No." Then they don't ask anything else. Basically,  that's it. I would expect them to ask more questions,” |
|  | Feeling pressured  [inductive] | This sub-category is to be used if a co-creator complains that interactions with healthcare workers feels pressured or stressful. | “It is really pressure that you have to just tell everything.” |
|  | Stigmatisation  [inductive] | This sub-category is to be used if a co-creator complains or presents a concern of feeling stigmatised when sharing information with healthcare workers. It is also to be used if a co-creator expresses a wish for the healthcare worker to be non-judgemental during the interaction. | “However, even here, I feel like it's difficult to get actual medical health if you're not perfect in the way. Like, I have chest pains, do you smoke? Do you drink? Do you do this? If you say yes, they say can you stop drinking and start walking, etcetera. I can have problems but I don't want to go to the doctor because I already know what they're going to tell me.” |
|  | Confidentiality  [inductive] | This sub-category is to be used if a co-creator complains or presents a concern that the conversation with the healthcare worker is not confidential. It is also to be used if a co-creator expresses a wish for the healthcare worker to not disclose information provided by patients. | “Because next thing you know, then it's all over the place, everybody knows and not even just sexual stuff in general.” |
|  | Tailored  [inductive] | This sub-category is to be used if a co-creator states that they expect healthcare workers to adapt their communication style to the needs of the patient. This includes considering the patient’s culture, communication style, past life experiences and feelings. This can also be expressed as a wish by the patient to communicate in a “German way” or use a communication style that is culturally appropriate. | “Because doctors here never give me what I want. It’s better to have someone that you know, and that person will know what to give you.” |
|  | Limited listening and time  [inductive] | This sub-category is to be used if a co-creator complains that the healthcare workers do not listen to them or there is not sufficient time in the consult. It is also to be used when co-creators express a wish that healthcare workers would listen more or they had more time with the healthcare worker. | “I feel like a lot of doctors work on a tight schedule, so it's not as if they have the time to sit there and listen to you and try to figure out what you're trying to say.” |
|  | Racist attitudes [inductive] | This sub-category is to be used if a co-creator explicitly complains that healthcare workers have a racist attitude or provide examples of where they were discriminated against based on race. Discrimination includes perceived and actual differences in how they were treated relative to German Caucasians, or those broadly referred to as “German”. | “I think what I would say, it's common because of these ancestors and what happened in the past is the level of nationalism and to a certain point, racism.” |
|  | Other | This sub-category is to be used if a co-creator has detailed a need or complaint that does not match the definitions provided in any of the other sub-categories, or is not clear the nature of the need or complaint being expressed. |  |
| System challenges | Wait time  [inductive] | This sub-category is to be used if a co-creator complains about the length of time they have to wait either at the clinic or to get an appointment. | “I stay in the waiting room more, of course, than in the doctor's room.” |
|  | Booking an appointment  [inductive] | This sub-category is to be used if a co-creator complains about the process of making an appointment, not related to the wait time. This can include challenges in getting hold of the clinic, or difficulties using the booking portal. | “The whole street is completely clean. You go there, you will not even get one appointment in there because it's not your closest doctor.” |
|  | Digitisation  [inductive] | This sub-category is to be used if a co-creator complains about the lack of digitization in the healthcare system. | “Maybe another challenge could be that doctors are not using technology. We have emails, we have WhatsApp, we have a lot of platforms that we could use for them to give us information or test results or updates that we can share on how we are feeling maybe with them. I feel like it's dumb for them to not trust email, Gmail, for example. They are very old-fashioned.” |
|  | Prescriptions  [inductive] | This sub-category is to be used if a co-creator complains about the challenges in getting a prescription or the process of getting medication at the pharmacy once they have a prescription, or more broadly about the challenges of getting medication or some other form of remedy. | “For one thing, here in Germany, it has been a lot more difficult to get a prescription for one thing and to get medicine. You don't get both at the same place.” |
|  | Insurance  [inductive] | This sub-category is to be used if a co-creator complains about needing insurance to receive care or complains about the process of securing insurance or getting their care covered by their insurance provider. | “Even the medication, there are a few medications that come under  the insurance, and most of them don't come under the insurance.” |
|  | Insufficient care  [inductive] | This sub-category is to be used if a co-creator complains about a lack of healthcare workers or services in Germany. This can include not being able to find a suitable doctor, clinics being understaffed or certain specialties or areas of medical care not being available in Germany. | “Just to mention that somehow [institution \| service: healthcare \| focus: primary_care], [institution \| service: healthcare \| focus: primary_care] is always on vacation when the majority of students need him.” |
|  | Test results  [inductive] | This sub-category is to be used if a co-creator complains about the process getting test results. This can include clinics not sending test results, or having to see the doctor directly to view results or not getting a personal copy of the test results. | “That's another thing. If they run tests on you, they tell you they're going to call you, and I think there's a list or something you're on.” |
|  | Navigating system  [inductive] | This sub-category is to be used if a co-creator complains about the complexity of the healthcare system or that they are not sure how to navigate it. This can include not knowing where to go for healthcare, or what the implications are for using a healthcare service, or more broadly, how the system operates. This also extends to a lack of information about the system or a wish for more information on how it works. | “I think for me, in terms of lack of information, because I arrived pretty late on campus. When I arrived, I didn't know who [institution \| service: healthcare \| focus: primary_care] was,I didn't know anything.” |
|  | Other | This sub-category is to be used if a co-creator has mentioned a system level challenge that does not match the definitions provided in any of the other sub-categories or when it is not clear what they are referring to. |  |
| Assumptions about responsibility | Primarily doctor  [inductive] | This sub-category is to be used when a co-creator explicitly states that the doctor is primarily responsible for safe and/ or effective communication in a doctor-patient setting. | “Mostly, healthcare professionals must be responsible” |
|  | Primarily patient  [inductive] | This sub-category is to be used when a co-creator explicitly states that the patient is primarily responsible for safe and/ or effective communication in a doctor-patient setting. | “For me, I think it's both. However, at the same time, I do think it's mostly the patient's role to provide information to the doctor.” |
|  | Primarily system/ governance/ institutional responsibility  [deductive] | This sub-category is to be used when a co-creator explicitly states that the healthcare system, institutions, or those in governance roles such as politicians are responsible for safe and/ or effective communication in a doctor-patient setting. | “I feel like the solution simply is just like having the German government and somehow making more doctors available to everybody.” |
|  | Shared responsibility not further specified  [inductive] | This sub-category is to be used when a co-creator states that the system, patient and doctor share equal responsibility or all play a role in ensuring safe and/ or effective communication in a doctor-patient setting without explicitly stating who has the largest share of the responsibility. | “I think I agree with both of them. I think it's about responsibility.” |
|  | Other | This sub-category is to be used when a co-creator specifies an assumption about who is responsible and does not fit any of the other sub-categories or is unclear. |  |
| Emotions whilst waiting for doctor | Fear or anxiety  [deductive] | This sub-category is to be used when a co-creator expresses feelings of apprehension, worry, or unease regarding their health condition, the upcoming medical examination, or the potential outcomes of their visit to the doctor. | “They're intimidating over there.” |
|  | Anger or frustration  [deductive] | This sub-category is to be used when a co-creator expresses feelings of irritation, annoyance, or dissatisfaction in relation to their experience while waiting to see the doctor. | “I don't have a problem with waiting. I have a problem with waiting, not knowing where I'm going or if I'm going to get the proper service or not.” |
|  | Sadness  [deductive] | This sub-category is to be used when a co-creator expresses feelings of sorrow, melancholy, or unhappiness during their wait to see the doctor. | “Feeling misunderstood” |
|  | Bored  [deductive] | This sub-category is to be used when a co-creator expresses feelings of disinterest or restlessness while waiting to see the doctor. | NA |
|  | Joy or happiness  [deductive] | This sub-category is to be used when a co-creator expresses feelings of delight, optimism, contentment, or pleasure while waiting to see the doctor. | NA |
|  | Other | This subcategory is to be used if a co-creator has reflected on an emotion that does not match the definitions provided in any of the other sub-categories or when the emotion is not clear. |  |
| Thoughts whilst waiting for doctor | Anticipation or expectations  [deductive] | This sub-category is to be used when a co-creator states that they think about possible health outcomes relating to their visit, or what the doctor might do or say. | “Is the doctor going to help me?” |
|  | Planning  [deductive] | This sub-category is to be used when a co-creator states that they think about what questions to ask the doctor, what information to share with the doctor or how to approach communication with their physician. | “Noting symptoms down.” |
|  | Time of appointment  [deductive] | This sub-category is to be used when a co-creator states that they think about when the doctor will see them. | “Am I going to wait for too long?” |
|  | Waiting area observations  [deductive] | This sub-category is to be used when a co-creator states that they think about the people or objects in their waiting area. | NA |
|  | Work or study  [deductive] | This sub-category is to be used when a co-creator states that they think about their work or study related tasks, which are not related to their health, topic of enquiry with the doctor or their appointment in general. | NA |
|  | Other | This sub-category is to be used if a co-creator has mentioned having thoughts whilst waiting that do not match the definitions provided in any of the other sub-categories or when the topic of the thought is not clear. |  |
| Retaining DHI A | Functionality  [deductive] | This sub-category is to be used if a co-creator states that a functional feature of DHI A should be retained. Functional features include elements that facilitate navigation within the platform, adaptability across multiple devices, the lesson based format, diary entries, and interactive elements like quizzes or feedback mechanisms. | “Good structure” |
|  | Content  [deductive] | This sub-category is to be used if a co-creators states that content from DHI A should be retained. Content from the web-app includes text and images that convey information from the orientation material, lessons, and flashcards. | “For me to say if the changes were implemented, then I think I would use, if not the whole app and maybe some lessons that could be especially useful, yes.” |
|  | Design  [deductive] | This sub-category is to be used if a co-creator states that a design element from DHI A should be retained. Design elements include colour palette, layout of widgets, font features, and other visual aesthetics. | NA |
|  | Handling data  [deductive] | This sub-category is to be used if a co-creator states that methods for how data are handled in DHI A should be retained. Data handling includes statements about what data is collected, how it is stored, and how it is used. | NA |
|  | Web-app  [deductive] | This sub-category is to be used if a co-creator states that the web-app aspect of DHI A should be retained. | NA |
|  | Other | This sub-category is to be used if a co-creator has mentioned an aspect of DHI A to retain that does not match the definitions provided in any of the other sub-categories or when it is not clear what they are referring to. |  |
| Modifying DHI A | Functionality  [deductive] | This sub-category is to be used if a co-creator states that a functional feature of DHI A should be dropped or is not fit for purpose for the patient preparedness tool. Functional features include elements that facilitate navigation within the platform, adaptability across multiple devices, diary entries, the lesson based format and interactive elements like quizzes or feedback mechanisms. | “They don't get feedback. It's just useless. It's just stressing.” |
|  | Content  [deductive] | This sub-category is to be used if a co-creators states that content from DHI A should be dropped or is not fit for purpose for the patient preparedness tool. Content from the web-app includes text and images that convey information from the orientation material, lessons, and flashcards. | “Yes, I think it's a bit content-heavy” |
|  | Design  [deductive] | This sub-category is to be used if a co-creator states that a design element from DHI A should be dropped or is not fit for purpose. Design elements include colour palette, layout of widgets, font features, and other visual aesthetics. | “Need easy to use application with minimalism and color-coding system.” |
|  | Handling data  [deductive] | This subcategory is to be used if a co-creator states that methods for how data are handled in DHI A should be changed. Data handling includes statements about what data is collected, how it is stored, and how it is used. | “Need a practical data collection tool” |
|  | Web-app  [deductive] | This subcategory is to be used if a co-creator states that the web-app medium of DHI A is not appropriate for the patient preparedness tool. | NA |
|  | Other | This subcategory is to be used if a co-creator has mentioned an aspect of DHI A to drop or modify that does not match the definitions provided in any of the other sub-categories or when it is not clear what they are referring to. |  |
| Utility of tool | No use  [inductive] | This sub-category is to be used if a co-creator states that they would not use the tool to help prepare for conversations with the doctor nor do they see others using the tool. | “also don't think I would be using it.” |
|  | One time use  [inductive] | This sub-category is to be used if a co-creator states that they would use the tool or they see other people using the tool only once and never again. | “Yes, I was just thinking while we were discussing that I feel it's a one-time thing.” |
|  | Important consultations  [inductive] | This sub-category is to be used if a co-creator states that they would use the tool or they see other people using the tool for only important consultations or for conversations with the doctor where they feel they need additional support. | “In that kind of scenario, maybe you're trying to convince them. In that case it might be useful, but other than that, it's a very low chance of using it.” |
|  | All consultations  [inductive] | This sub-category is to be used if a co-creator states that they would use the tool or they see other people using the tool to help prepare for all consultations. | “Yes, I would do it too” |
|  | Conditional use  [inductive] | This sub-category is to be used if a co-creator states that they would use the tool if it has certain features or that they would use it sometimes. | “Yes. I would depending on the adaptations.” |
|  | Other | This sub-category is to be used if a co-creator has mentioned a pattern of usage that does not match the definitions provided in any of the other sub-categories or when it is not clear what they are referring to. |  |

Table 2. Table of the codes from the level two parent categories and definitions of each.

| L1 Category | L2 Category | Code | Definition | Example |
| --- | --- | --- | --- | --- |
| Preparation | Preparation timing | On the day  [deductive] | This sub-category is to be used if a co-creator states that they prepare for the consultation on the day of the appointment. | “Yeah but on the point of whether it takes long, I think it's usually like in the morning off” |
|  |  | Before the day  [deductive] | This sub-category is to be used if a co-creator states that they prepare for the consultation before the day of the appointment. | “I think mostly like the day before.” |
|  |  | Other | This sub-category is to be used if a co-creator has mentioned a time for preparing, which does not match the definitions provided in any of the other sub-categories or it’s not clear when they prepare. |  |
|  | Preparation location | In the clinic  [deductive] | This sub-category is to be used if a co-creator states that they prepare for the consultation whilst waiting in the clinic. | NA |
|  |  | At home  [deductive] | This sub-category is to be used if a co-creator states that they prepare for the consultation at home. | “however, the preparation starts way earlier when I just like to search about what symptom it could be or what disorder or something? Is it important? It starts a little earlier when I'm in my house” |
|  |  | On the way  [deductive] | This sub-category is to be used if a co-creator states that they prepare for the consultation on the way to the clinic. | “On the way.” |
|  |  | Other | This sub-category is to be used if a co-creator has mentioned a location used for preparing, which does not match the definitions provided in any of the other sub-categories or it’s not clear where they prepare. |  |
|  | Preparation method | Taking notes  [deductive] | This sub-category is to be used if a co-creator states that they prepare for the consultation by taking notes either digitally or using pen and paper. | “Usually, I write down everything I have to say to the doctor.” |
|  |  | Mental review  [deductive] | This sub-category is to be used if a co-creator states that they prepare for the appointment by only thinking about what they would like to say. | “I did mentally prepare because it wasn't that many things.” |
|  |  | Language translation  [deductive] | This sub-category is to be used if a co-creator states that they translate points that they would like to address with the physician prior to the consultation. | “I just prepare with Google Translate.” |
|  |  | Research  [deductive] | This sub-category is to be used if a co-creator states that they prepare for the appointment by searching for resources related to their health concern or topic of discussion with the doctor. | “Looking up symptoms online is another thing, but I don't know if you want to go over that or not.” |
|  |  | No preparation  [inductive] | This sub-category is to be used if a co-creator states that they do not prepare for the appointment. This also includes instances where the co-creator states that they just know what to say. | “I didn’t prepare for it.” |
|  |  | Other | This sub-category is to be used if a co-creator has commented on a method to preparing, which does not match the definitions provided in any of the other sub-categories or when their preparation method is not clear. |  |
|  | Preparation reason | Prompt doctor  [inductive] | This sub-category is to be used if a co-creator states that the reason for preparing is to help the doctor diagnose or treat them. Prompting the doctor could relate to giving them cues to ask questions, helping the doctor think of possible causes or treatments to their healthcare concern. Typically, patients will mention wanting to understand their symptoms and/ or treatment options to query the possibility of a causal factor, or effectiveness of a treatment option. | “and then so that I just give them like go prompt or a cue, like to start asking me questions.” |
|  |  | Efficient communication  [inductive] | This sub-category is to be used if a co-creator states that the reason for preparing is to manage the efficient flow of information during the consultation. Typically, patients will mention wanting to ensure all content is covered and/ or all relevant questions are asked of the doctor within the time available. | “If you forget something, it’s bad because your next appointment is going to be in a while.” |
|  |  | Psychological safety  [inductive] | This sub-category is to be used if a co-creator states that the reason for preparing is to feel less anxious and have greater confidence when speaking to the doctor. Typically, patients will mention wanting to feel mentally and emotionally prepared for the appointment. | “to look the right way because I want to be taken seriously. If I don't prepare myself mentally and physically, they won't take me seriously anyway.” |
|  |  | Other | This sub-category is to be used if a co-creator has detailed a reason to preparing, which does not match the definitions provided in any of the other sub-categories or when their reason for preparation is not clear. |  |
|  | Preparation assistance | Friends  [deductive] | This sub-category is to be used if a co-creator has stated that friends are consulted in helping prepare for the appointment. This can include speaking to friends prior to the appointment and going to the clinic with friends. | “At first I got sick and luckily I had a friend who didn't just take me to  [institution \| service: healthcare \| focus: primary_care], but we went to this hospital in [places \| region: Germany \| location: local].” |
|  |  | Partner or family  [deductive] | This sub-category is to be used if a co-creator has stated that a partner or family member(s) are consulted in helping prepare for the appointment. This can include speaking to the support person prior to the appointment and going to the clinic with the support person. | “but I had to go first to her with my [disclosure_control] who speaks German so he could ask her if she can speak English to me. Then she said, yes I can speak English and then he asked her in German can she speak English with me.” |
|  |  | No assistance  [deductive] | This sub-category is to be used if a co-creator explicitly states that no one else is consulted in the preparation process or provides accompaniment for the clinic visit. | NA |
|  |  | Other | This sub-category is to be used if a co-creator has stated the role of others in helping prepare, which does not match the definitions provided in any of the other sub-categories or when it is not clear if or who provides preparation assistance. |  |
|  | Other | - | This sub-category is to be used if a co-creator has discussed preparation for primary care consults, which do not match the definitions provided in any of the other sub-categories or it’s not clear what aspect of preparation they are discussing. |  |
| Needs for a digital tool | Feature inclusion | Patient empowerment  [deductive] | This sub-category is to be used when a co-creator explicitly expresses a need for tools or resources that empower them in a healthcare setting. This can include tools that increase confidence, self-efficacy, power and feeling in control. | NA |
|  |  | Patient education  [inductive] | This sub-category is to be used when a co-creator expresses a need for tools or resources that provide them with knowledge about their health condition, symptoms, medical procedures, or rights as a patient. It includes features aimed at educating patients on various aspects of healthcare to facilitate informed decision-making and effective communication with healthcare providers. | “So you get a few options about the symptoms that have and then present an overview of your situation, whether you should visit the hausarzt or not, and if you feel it is getting worse, then you can go to the Hausarzt.” |
|  |  | Appointment management and clinic search  [inductive] | This sub-category is to be used when a co-creator requests for features related to scheduling appointments or searching for clinics online. It encompasses functionalities such as online appointment portals, searching for clinics that can meet language needs, calendar integration, and appointment reminders to facilitate appointment scheduling. | “or some feature where you can make your appointment, for example, or where you can see the doctor etc., then I  would use it.” |
|  |  | Online accessibility to healthcare  [inductive] | This sub-category is to be used when a co-creator requests for digital modes of receiving health care. This includes online consultations and electronic prescriptions. | “If it had, maybe not the office hours, but maybe talk hours with the doctor then maybe,  in that case, I would be using it.” |
|  |  | Communication preparation tools  [inductive] | This sub-category is to be used when a co-creator identifies the need for communication tools to facilitate interaction between patients and healthcare providers. It includes tools for preparing for doctor visits, and guidance on effective communication strategies to enhance patient-provider interaction and support. | “We had problems going to the Hausarzt explaining our state, like giving an explanation”  “to the Hausarzt about our state, our symptoms. That was the main problem. If this app kind of helps in all of this, the communication between us and the doctors, then we can be better.” |
|  |  | Health monitoring and management  [inductive] | This sub-category is to be used when a co-creator requests for tools and functionalities related to monitoring, self-diagnosing, and managing their health. It encompasses features such as symptom tracking tools, monthly health reports, medication reminders, and access to personal health records. | “After the visit, upload their file to the app, so the patient can also have access to their file information” |
|  |  | Video based content  [inductive] | This sub-category is to be used if a co-creator has requested the use of videos to present the content. | “I think that's what they showed today with the videos and stuff and the types. That could be useful. I would definitely look into that. I feel like that has some value. It would make me more confident, and feel safer,” |
|  |  | Text based content [deductive] | This sub-category is to be used if a co-creator has requested that content be presented in text. | NA |
|  |  | Native app  [inductive] | This sub-category is to be used if a co-creator requests that the tool be made available as a native app. | “I think it's nice to keep the app on the phone. Whenever you feel like it, you can just go back to it.” |
|  |  | Web-app  [inductive] | This sub-category is to be used if a co-creator requests that the tool be accessible through a web-browser. | “It could be a centralized website where the lessons are, and there could be that kind of outreach anyway.” |
|  |  | Interactivity  [inductive] | This sub-category is to be used if a co-creator requests that the tool provide feedback or interact with the user based on responses provided by the user. This can include a wish that the tool adapt the design or features based on the needs of the user, respond to user input of open ended items or incorporating a chatbot. | “interactive sessions,” |
|  |  | Other | This sub-category is to be used if a co-creator has requested a feature to be included in the digital tool that does not match the definitions provided in any of the other sub-categories or when the need stated is not clear. |  |
|  | Feature exclusion | Patient empowerment  [deductive] | This sub-category is to be used when a co-creator explicitly requests that the tool not look to address patient empowerment. |  |
|  |  | Patient education  [deductive] | This sub-category is to be used when a co-creator requests that the tool not look to provide tools or resources that increase knowledge about their health condition, symptoms, medical procedures, or rights as a patient. | “The reason we visit the doctor is the doctor gives us an overview rather than reading everything.” |
|  |  | Appointment management and clinic search  [deductive] | This sub-category is to be used when a co-creator requests that the tool not have features related to scheduling appointments, managing appointments online or searching for appropriate clinics. |  |
|  |  | Online accessibility to healthcare  [deductive] | This sub-category is to be used when a co-creator requests that the tool not have features related to online consultations or receipt of electronic prescriptions. |  |
|  |  | Communication preparation tools  [deductive] | This sub-category is to be used when a co-creator requests that the tool not have features related to doctor-patient communication nor those to help patients prepare for conversations with health care workers. | “In a way that it is, it's just communication and stuff like that, maybe that's particularly me, because I'm a bit disillusioned with the entire medical system and stuff, I don't see a value for me.” |
|  |  | Health monitoring and management  [deductive] | This sub-category is to be used when a co-creator requests that the tool not have features related to monitoring, self-diagnosing, and managing their health. |  |
|  |  | Video based content  [deductive] | This sub-category is to be used if a co-creator has specified that they do not want content presented in videos. | “I wouldn't watch videos.” |
|  |  | Text based content  [deductive] | This sub-category is to be used if a co-creator has specified that they do not want content presented in text. | “No one would want to read it if you are sick.” |
|  |  | Native-app  [deductive] | This sub-category is to be used if a co-creator requests that the tool not be developed as a native app. | “Can I add? One more thing is that people are not always sick. They don't need to have the app or use it like constantly.” |
|  |  | Web-app  [deductive] | This sub-category is to be used if a co-creator requests that the tool not be developed as a web-based app. | NA |
|  |  | Interactivity  [deductive] | This sub-category is to be used if a co-creator requests that the tool not provide feedback interact with the user based on responses provided by the user. This can include a request for a static design, no response from the tool to user input of open ended items or excluding a chatbot. | NA |
|  |  | Other | This sub-category is to be used if a co-creator requests that the tool not have a given feature but it does not match the definitions of any of the other subcategories or is not clear what they are referring to. |  |
|  |  |  | This sub-category is to be used if a co-creator specifies a need for a digital tool that does not match the definitions of the other sub-categories or is not clear. Comments associated with intended target groups of the digital tool should also be coded here. |  |
|  | Other | - | This sub-category is to be used if a co-creator specifies a need for a digital tool that does not match the definitions of the other sub-categories or is not clear if it should be included or excluded. Comments associated with intended target groups of the digital tool should also be coded here. |  |
