# Supplementary material for: Cocreating a Digital Patient Preparedness Tool for International Students Accessing Primary Care in Germany: A Convergent Methods Study Within the Health CASCADE Network
Source: JMIR Hum Factors. 2026 Sep 14;13:e87519. doi: 10.2196/87519 (PMC13574332; doi:10.2196/87519)
Supplement: Supplementary file 4 [file humanfactors-v13-e87519-s004.docx]

# Quality of coding frame

Table 1. Table of the reliability and validity assessments for each iteration of the coding frame. Higher percentages for reliability indicate higher reliability. Lower percentages for validity indicate higher validity.

| Coding frame ID | Size of sample | Inter-rater consistency | Validity |
| --- | --- | --- | --- |
| 2024_05_01 | 45.95% | 50.27% | 43.26% |
| 2024_05_18 | 27.07% | 74.56% | 17.31% |
| 2024_05_31 | 3.82% | 75.96% | 5.89% |
| 2024_06_05 | 4.20% | 74.44% | 4.2% |

Table 2. Table presenting the change in inter-rater consistency and validity between the first and last iterations of the coding frame. Higher percentages for reliability indicate higher reliability. Lower percentages for validity indicate higher validity.

| Category | 2024_05_01 | | 2024_06_05 | |
| --- | --- | --- | --- | --- |
|  | Inter-rater consistency | Validity | Inter-rater consistency | Validity |
| Needs and complaints | 42.42% | 43.93% | 88.89% | 1.98% |
| System challenges | NA | NA | 83.33% | 1.41% |
| Assumptions about responsibility | 40% | 33.33% | NA | 3.57% |
| Preparation | 76.92% | 30.77% | NA | 7.58% |
| Emotions whilst waiting for the doctor | 100% | 0% | NA | 11.54% |
| Thoughts whilst waiting for the doctor | 50% | 50% | NA | 3.57% |
| Needs for a digital tool | 56.25% | 64.58% | 50% | 7.41% |
| Retaining DHI A | 26.67% | 13.33% | NA | 0% |
| Modifying DHI A | 16.67% | 16.67% | 50% | 2.60% |
| Utility of tool | NA | NA | 100% | 7.69% |
