# Supplementary material for: Cocreating a Digital Patient Preparedness Tool for International Students Accessing Primary Care in Germany: A Convergent Methods Study Within the Health CASCADE Network
Source: JMIR Hum Factors. 2026 Sep 14;13:e87519. doi: 10.2196/87519 (PMC13574332; doi:10.2196/87519)
Supplement: Supplementary file 5 [file humanfactors-v13-e87519-s005.docx]

# Co-creator experiences and behaviour

Figure 1. Heatmap of typical visits to the clinic with respect to arranging the appointment and assistance sought.

Figure 2. Heatmap of typical visit to the clinic with respect to waiting time and location.

Figure 3. Heatmap of typical visit to the clinic with respect to waiting room presence and waiting location.

Figure 4. Heatmap of mobile phone use in clinic with respect to clinic policy and mobile data signal strength.

Figure 5. Stacked bar chart on who is responsible for effective communication in a healthcare setting.

Figure 6. Heatmap of feeling misunderstood and not being listened to when communicating with doctors.

Figure 7. Bar graph of assumptions on responsibility.

Figure 8. Bar graph of needs and complaints in healthcare settings.

Figure 9. Bar graph of challenges in German healthcare system.

Figure 10. Bar graph of emotions whilst waiting.

Figure 11. Bar graph of thoughts whilst waiting.

Figure 12. Bar graph of preparation methods.

Figure 13. Bar graph of preparation timing.

Figure 14. Bar graph of preparation location.

Figure 15. Bar graph of preparation reasons.
