# Supplementary material for: Cocreating a Digital Patient Preparedness Tool for International Students Accessing Primary Care in Germany: A Convergent Methods Study Within the Health CASCADE Network
Source: JMIR Hum Factors. 2026 Sep 14;13:e87519. doi: 10.2196/87519 (PMC13574332; doi:10.2196/87519)
Supplement: Supplementary file 6 [file humanfactors-v13-e87519-s006.docx]

# Co-creator needs for a digital health intervention

Figure 1. Bar graph of DHI A features to retain.

Figure 2. Bar graph of DHI A to modify.

Figure 3. Bar graph of features to include in a DHI.

Figure 4. Bar graph of features to exclude in a DHI.

Figure 5. Bar graph of comments regarding utility of a patient preparedness tool.
