# Supplementary material for: Cocreating a Digital Patient Preparedness Tool for International Students Accessing Primary Care in Germany: A Convergent Methods Study Within the Health CASCADE Network
Source: JMIR Hum Factors. 2026 Sep 14;13:e87519. doi: 10.2196/87519 (PMC13574332; doi:10.2196/87519)
Supplement: Supplementary file 7 [file humanfactors-v13-e87519-s007.docx]

# Behaviour change intervention specifications

In this study, the target behaviour identified was patient preparedness, conceptualised as the act of taking notes, listing symptoms, and organising concerns prior to a general practitioner consultation. Co-creators commonly reported engaging in this behaviour as a means of structuring the conversation, prompting physician engagement, and ensuring they voiced their health-related needs effectively.

To support patient preparedness by means of digital medicine and health care in the internet age, several levers for change were identified, mapped to the COM-B components of capability, opportunity, and motivation. To build capability, the intervention should offer some form of communication training, but not in the form of structured lessons, which many co-creators found cognitively demanding and emotionally taxing. Instead, co-creators preferred more digestible tools, such as a concise checklist, to guide what to prepare ahead of a consultation. To create opportunities for preparation, encouragement from physicians was seen as essential. Several co-creators reported that when doctors appeared disengaged, they felt less inclined to prepare. Appointment confirmation messages or clinic signage prompting patients to prepare could serve as effective cues. In terms of motivation, co-creators indicated that the tool’s perceived value would increase if it also addressed broader system-level frustrations. Requests included appointment scheduling features, clinic directories, and digital access to test results. Additionally, co-creators proposed interactive elements, particularly a chatbot that could simulate a consultation or offer preparation feedback, as more engaging and acceptable behaviour change techniques.

Beyond behavioural components, co-creators also pointed to broader system-level challenges that a digital tool could help address. They noted that preparation alone was insufficient to overcome structural frustrations, such as appointment scheduling difficulties and lack of clear clinic information. As a result, integrating additional functions, such as scheduling support, symptom tracking, clinic directories, or access to digital documentation, could increase tool uptake and sustained engagement. This is consistent with prior findings indicating that adoption of digital health interventions is often driven by breadth of features and perceived practical value [83,84].

Table 4. Mapping co-creation findings from international students in Germany to COM-B determinants, behaviour change techniques, and proposed features for a digital patient preparedness intervention.

| Specification | Evidence from co-creation workshops |
| --- | --- |
| Target audience | International students |
| Target behaviour | Patient preparedness. Preparation behaviours were reported as a way to structure consultations, prompt physicians, and psychologically prepare for the encounter |
| Capability (Build capabilities for preparation behaviour) | Build patient capability through communication training; checklist with prompts for symptoms, questions, and concerns |
| Opportunity (Create opportunities for patient engagement) | Create opportunity for patient engagement through encouragement from physicians for patients to prepare |
|  | Create opportunities for use by integrating the intervention into existing tools or resources |
| Motivation (Motivate patient preparedness) | Increase motivation to engage in patient preparation using Chatbot or AI-supported consultation simulation |
|  | Increase motivation to engage in tool by incorporating features that provide information on clinics, and access to patient rights information |
|  | Increase motivation to engage in tool by integrating appointment booking, digital health services, or clinic resources |
| Behaviour change technique (BCT 1.4 –Action planning) | Co-creators preferred concise preparation prompts (i.e. a checklist of when, where and how behaviour should be performed) rather than structured lessons |
| Behaviour change technique (BCT 8.1 – Behavioural practice) | Co-creators requested interactive components (i.e. a Chatbot) to guide preparation and communication |
| Broader support | Co-creators reported difficulties navigating the healthcare system; intervention could be supported through university orientation programmes |
